# The effects of H3N2 swine influenza virus infection on TLRs and RLRs signaling pathways in porcine alveolar macrophages

**DOI:** 10.1186/s12985-015-0284-6

**Published:** 2015-04-14

**Authors:** Jinqiu Zhang, Jinfeng Miao, Jibo Hou, Chengping Lu

**Affiliations:** National Research Center for Veterinary Vaccine Engineering and Technology of China, Jiangsu Academy of Agricultural Sciences, Nanjing, 210014 China; College of Veterinary Medicine, Nanjing Agricultural University, Nanjing, 210095 China

**Keywords:** H3N2, Swine influenza virus, TLRs, RLRs, Alveolar macrophage

## Abstract

**Background:**

Swine influenza is an economically important respiratory disease of swine resulting from infection with influenza A virus. Swine influenza virus (SIV) becomes the focus as pigs have been hypothesized to serve as an intermediate host for the adaptation of avian influenza viruses to humans or as mixing vessels for the generation of genetically reassortant viruses. The ability of the innate immune system to detect and respond to pathogens is important for survival. Therefore, there is a critical need to evaluate the immediate response to viral infection, especially the role of the toll-like receptors (TLRs) and RNA helicase RIG-I-like receptors (RLRs) innate immunity signaling pathways in H3N2 swine influenza virus infection.

**Method:**

In this study, porcine alveolar macrophages (PAMs) were obtained from porcine lungs and were infected with SIV at a multiplicity of infection (MOI) of 5 *in vitro*. The changes of the related receptors, signaling proteins and effector molecules of TLRs and RLRs signaling pathways post H3N2 virus infection of PAMs were quantified by Real-time quantitative RT-PCR and western blotting.

**Results:**

The results showed that H3N2 SIV infection significantly increased mRNA expression of TLR-3, TLR-7, RIG- I and MDA5 after 4 hpi (P *<* 0.05). Western blotting showed that the protein levels of TLR-3, TLR-7 and RIG-I also had a significantly increase after PAM exposed to virus. A significant change of MyD88, MAVS, IRF-3 and IRF-7 mRNA expression were present at 8 hpi. More than a 4-fold increase was induced for TNF-α and IL-1β mRNA expression. And the concentration of TNF-α and IL-1β peaked at 12 and 24 hpi, respectively. IFN-α, IFN-β mRNA and protein levels increased after SIV infection and significant differences was observed at 8, 12 and 24 hpi.

**Conclusion:**

These results indicate that H3N2 swine influenza virus infection significantly influences the expression of the receptors, adapter proteins and downstream effector molecules of RLRs and TLRs signaling pathways. This study enhances our understanding of innate immunity signaling pathways in PAM anti-infection of H3N2 SIV.

## Introduction

Swine influenza is common in pig populations worldwide. In infected pigs, disease is characterized by fever, lethargy, sneezing, coughing, difficulty breathing and decreased appetite. In sows, it may cause abortions. Although mortality is usually low, swine influenza infection may result in poor growth, weight loss, and immunosuppression resulting in secondary infection by other pathogens, causing economic loss to the pig industry [1,2].

Influenza virus is a negative (−) sense ssRNA virus that infects a diverse range of vertebrates. Of the 3 genera of influenza viruses (A, B, C) that cause human flu, influenza A is most frequently diagnosed in swine, influenza C is rare and influenza B has not been reported in pigs. Among influenza A subtypes, H1N1, H1N2 and H3N2 are known to cause disease in pigs [3-6]. Recently, swine-origin influenza A H3N2 variants were determined to be triple reassortants, containing genes from human, swine and avian lineages. This indicates that pigs play an important role in influenza ecology and serve as “mixing vessels” for new influenza strains [6,7]. Therefore, there is a critical need to better understand the pathogenic mechanisms of these viruses.

The innate immune system is the first host defense against viral infection [8]. In mammalian target cells, several innate immune pathways are critical for protection against RNA viruses and depend on the recognition of viral signature molecules such as ssRNA, dsRNA and envelope proteins. The cell receptors that trigger these pathways are located in different cell compartments such as the cell surface, endosomes and cytoplasm. Toll-like receptors (TLRs) and RNA helicase RIG-I-like receptors (RLRs) are two important classes of cell receptors. TLR (mainly TLR-3, TLR-7, TLR-8) and RLR [retinoic acid-inducible gene I (RIG-I) and melanoma differentiation-associated gene 5 (MDA5)] mediated signaling and transcriptional events initiated by various target cells (including macrophages, dendritic, natural killer and respiratory epithelial cells, as well as others) are critical in responding to influenza A infection in humans and in animal models [9-11]. For example, Wei et al. demonstrated that duck MDA5 is an important receptor for inducing antiviral activity in the host immune response in ducks [12]. The research of Ramírez-Martínez et al. showed that the pdm A/H1N1 viral strain could increase the production of inflammatory mediators by inhibiting SOCS-1 and modifying the expression of antiviral immunity-related genes, including RIG-I, in human macrophages [11]. Little is available regarding the effects of these signaling pathways in swine influenza infection in pigs. Different hosts may have different responses to the same virus challenge [13]. Gao et al. indicated that in swine macrophages infected with H1N1, MAP kinase may activate NF-κB through the induction of RIG-I, which leads to the induction of IFN-β [14]. Dobrescu et al. found that TLR-3, RIG-I and IFNβ were important in cellular and tissue immune responses in PRRSV/SIV co-infection [15]. How these pathways are coordinated is unknown.

Influenza viruses mainly infect respiratory epithelial cells in humans and animals. In recent years, it has been shown that alveolar macrophages may play an important role in influenza A infection. Seo et al. found, in a pig model, that human H3N2 influenza virus can infect alveolar macrophages and reside in the respiratory tract without causing apoptosis; however, highly inflammatory cytokines are induced [16]. Kim et al. showed that alveolar macrophages are indispensable for controlling H1N1 influenza virus infection in pigs using an *in vivo* alveolar macrophage depletion model [17]. As versatile immune cells, macrophages express TLRs and RLRs and have roles in innate and adaptive immunity [18]. It has previously been reported that different cell types induced different innate immunity responses to various viral strains [13]. This knowledge led to our interest in studying changes in the innate signaling pathways mediated by these 2 receptor families in primary porcine alveolar macrophages (PAMs) after H3N2 swine influenza virus (SIV) infection. Herein, we report the replication of H3N2 SIV in primary PAMs and the influence of infection on receptors, signaling proteins and effector molecules of TLRs and RLRs signaling pathways.

## Materials and methods

### Ethics

PAMs were obtained from 3–6 week old healthy pigs with no clinical symptoms or evidence of influenza or other respiratory or systemic diseases based on serological and PCR testing. The animal protocols were approved by the Science and Technology Agency of Jiangsu Province. All efforts were made to minimize animal suffering.

### Virus and titration

Swine influenza virus (SIV, A/Swine/Shandong/3/2005, H3N2 subtype) was propagated in the allantoic membranes of 9-day-old, embryonated, specific-pathogen-free chicken eggs or in Madin–Darby canine kidney cells (MDCK (NBL-2)). Viral stocks were titrated on MDCK cells. Briefly, virus was serially diluted with serum-free DMEM (Invitrogen Life Technology, Shanghai, China) supplemented with 1 μg/mL tosylsulfonyl phenylalanyl chloromethyl ketone (TPCK)-trypsin (Sigma). 100 μL of inoculum was overlaid onto monolayers of MDCK cells in 96-well plates and incubated for up to 120 h. The 50% tissue culture infectious dose (TCID_50_) was calculated by the Reed-Muench method.

### Porcine alveolar macrophage isolation and culture conditions

PAMs were obtained by lung lavage from 3 pigs for each independent experiment as previously described [19]. The collected cells were suspended in RPMI-1640 medium supplemented with 10% FBS, 100 IU/mL penicillin and 100 μg/mL streptomycin and the cell concentration was adjusted to 2 × 10^6^/mL. The isolated cells were incubated at 37 C in 5% CO_2_ overnight prior to assay.

### Exposure of cells to virus and samples collected

PAMs were infected with SIV at a multiplicity of infection (MOI) of 5 (Data not shown). Mock-treated cells (controls) received virus-free culture medium supplemented with 1 μg/mL TPCK-trypsin. All experiments were performed in triplicate. Macrophages were harvested for RNA isolation at 0, 2, 4, 6, 8, 12, and 24 hpi. Culture supernatants at different time points were collected for cytokine measurement. For virus titration assays, both the supernatants and cells were harvested at 0, 4, 8, 12, 24, 36 and 48 hpi. After freezing and thawing 3 times, the supernatants were collected by centrifugation (10,000 × g, 15 min, 4 C) for TCID_50_ assay.

### RNA extraction and RT-PCR

#### RNA extraction

Total RNA was extracted from PAMs harvested at 0, 2, 4, 6, 8, 12 and 24 hpi using TRIZOL reagent (TaKaRa, Dalian, China) according to the manufacturer’s protocols. RNA was quantified by measuring absorbance at 260 nm (Eppendorf Biophotometer). The ratios of absorption (260/280 nm) of all samples were between 1.8 and 2.0. Aliquots of RNA were subjected to electrophoresis through a 1.4% agarose formaldehyde gel to verify their identities.

#### Real-time quantitative RT-PCR

Synthesis of first strand complementary DNA (cDNA) was performed with reverse transcriptase and Oligo(dT)_18_ primer (TaKaRa, Dalian, China), according to the manufacturer’s instructions. The final volume of 20 μL contained 10 units of AMV reverse transcriptase, 1 mM dNTP mixture (TaKaRa, Dalian, China), 20 units of recombinant RNasin ribonuclease inhibitor (TaKaRa, Dalian, China), and 50 pmol of Oligo(dT)_18_ primer. After incubation (42°C, 60 min), the mixture was heated (95°C, 5 min). An aliquot of the cDNA sample was mixed with 25 μL SYBR® Green PCR Master Mix (TaKaRa, Dalian, China) in the presence of 10 pmol of each forward and reverse primer for TLR-3, TLR-7, TLR-8, MyD88, TRIF, TRAF3, TNF-α, IL-1β, RIG-I, MDA5, MAVS, IRF-3, IRF-7, IFN-α and IFN-β (Table 1) and subjected to PCR under standard conditions (43 cycles). As an internal control, the same RT products were subjected to PCR in the presence of a second pair of primers specific to β-actin. All primer sequences were synthesized by Invitrogen Biological Company (Shanghai, China). Mixtures were incubated in an ABI Prism 7300 Sequence Detection System (Applied Biosystems) programmed to conduct one cycle at 95°C for 10 min and 43 cycles at 95°C for 15 s and 62°C for 1 min. Results (fold changes) were expressed as 2^−ΔΔCt^ with ΔΔCt = (Ct ij − Ct β-actin j) − (Ct i1 − Ct β-actin1), where Ct ij and Ct β-actin j are the Ct for gene i and for β-actin in a sample (named j), and where Ct i1 and Ct β-actin1 are the Ct in sample 1, expressed as the standard. The 0 h group is determined as standard, thus resulting in a relative expression of 1 = 2^0^ at this time point [20,21].

**Table 1:** **Sequences of oligonucleotides used for PCR**

**Target gene**	**Accession number**	**Orientation**	**Primers sequence (5′-3′)**
β-actin	DQ845171.1	Forward Reverse	GACATCAAGGAGAAGCTGTGC TGAAGGTAGTTTCGTGGATGC
TLR-3	DQ266435.1	Forward Reverse	AAAACCAGCAACACGACT TTGGAAAGCCCATAAAGA
TLR-7	NM_001097434.1	Forward Reverse	GGCAAGTAGAGGACAT GGTAGACCCTGAACAT
TLR-8	AB092975.1	Forward Reverse	CGGCACCAGAAGAACG GGCAGGTCAGGAGCAA
MyD88	EF198416.1	Forward Reverse	CGTCGGATGGTAGTGG TGATGAACCGCAGGAT
TRIF	KC969185.1	Forward Reverse	ACCAAATGTCTGGACCCGATGA CCCGAGCAGTGTCCTGAAAGTA
TRAF3	XM_001927400.4	Forward Reverse	GTGTCAAGAAGGCATCG CCTCAAACTGGCAATCA
TNF-α	NM_214022.1	Forward Reverse	CGTTGTAGCCAATGTCA TAGGAGACGGCGATGC
IL-1β	NM_214055.1	Forward Reverse	AGTGATGGCTAACTACGG TCTCCACTGCCACGAT
RIG-I	EU126659.1	Forward Reverse	ATCCCAGCAACGAGAA GCCACGTCCAGTCAAT
MDA5	EU006039.1	Forward Reverse	CCTACGTCCTGGTTGC GATGGGTTGTCCTTGC
MAVS	AB287431.1	Forward Reverse	ATAGCCAGCCTTTCTCGG TAGCCTCAGTCTTGACCTCTTC
IRF-3	NM_213770.1	Forward Reverse	AGAAGCATTGCGTTTAGC TCACGGACTCCCAGGTT
IRF-7	NM_001097428.1	Forward Reverse	CGACTTCGGCACCTTCT GAGGACACGCCTTCACG
IFN-α	JQ839262.1	Forward Reverse	TCACAGAGTCACCCACC CATTTGTGCCAGGAGC
IFN-β	GQ415073.1	Forward Reverse	ACCAACAAAGGAGCAG TTTCATTCCAGCCAGT

### Total protein extraction and western blotting

Total protein extracts were prepared from cell samples (macrophages harvested at 12 and 24 hpi) using commercial kits (Pierce, USA). Protein concentrations were determined by bicinchoninic acid (BCA) assay and the samples were stored at −70°C until analyzed.

Cells were lysed in RIPA lysis buffer (1% Triton X-100, 0.25% deoxycholate (DOC), 0.05% SDS, 50 mM Tris pH8.0, 150 mM NaCl and 50 mM NaF) containing proteinase inhibitor cocktails (Roche Applied Science, Mannheim, Germany) for 30 min at 4°C. The concentration of protein in the lysates was assessed with the BCA protein assay reagent (Pierce Biotechnology, IL, USA). Cell lysates were analyzed by SDS-PAGE, and proteins were transferred to PVDF membranes (Millipore, MA, USA). Membranes were subsequently incubated with a primary antibody of RIG-I, TLR-3, TLR-7, MAVS and MyD88 (Cell Signaling Technology) followed by horseradish peroxidase (HRP)-conjugated secondary antibody (Bethyl Laboratories, Inc., TX, USA). Signals were detected using an Immobilon Western Chemiluminescent HRP Substrate Kit (Millipore, Shanghai, China).

### Cytokine measurement

Cytokines (TNF-α, IL-1β, IFN-α and IFN-β) were measured from supernatants (harvested at 0, 2, 4, 6, 8, 12 and 24 hpi) by radioimmunoassay. Commercial kits were purchased from the Institute of Radiation of Science and Technology Development Center of the General Hospital of People’s Liberation Army (Beijing, China). The assay was conducted according to the manufacturer’s manual.

### Statistical analyses

All statistical procedures, means, and standard errors of the means were computed using statistical software SPSS16.0. Data are expressed as means ± SEM. Differences were evaluated by using one-way analysis of variance (ANOVA) and considered significant at P < 0.05.

## Results

### Virus titer measurement of SIV propagated on PAM

**Figure 1:**
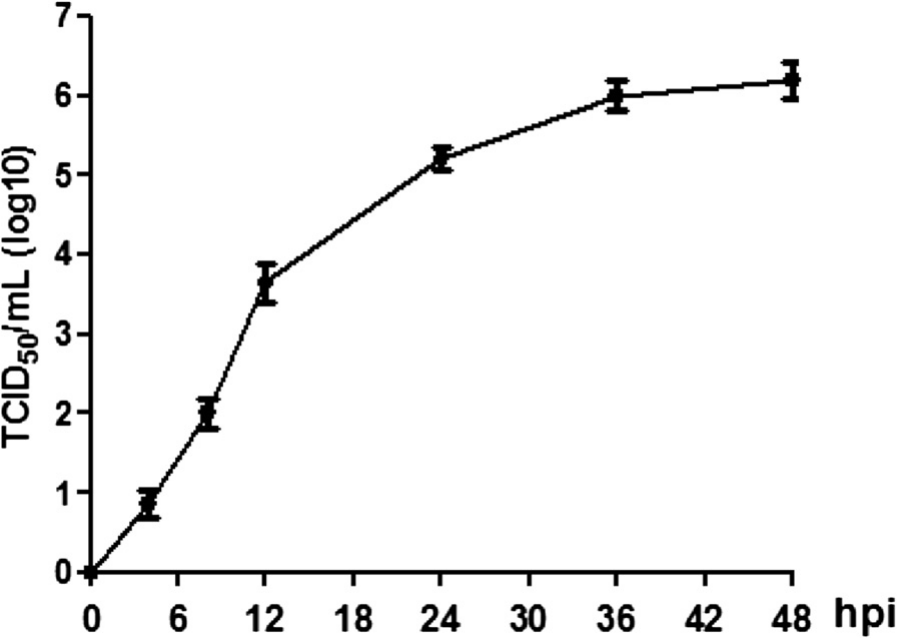
Virus titer measurement of SIV propagated on PAMs. PAMs were infected with SIV (A/Swine/Shandong/3/2005) at a multiplicity of infection of 5. Both the supernatant and cells were harvested at 0, 4, 8, 12, 24, 36 and 48 hpi. After freezing and thawing 3 times, the supernatants were collected by centrifugation (10,000 × g, 15 min, 4 C) for TCID_50_ assay. Results are representative of 3 independent experiments.

To analyse viral growth kinetics in PAMs, cells were infected with SIV (A/Swine/Shandong/3/2005) at a MOI of 5. The data showed that SIV (A/Swine/Shandong/3/2005) replicated rapidly in PAMs from 8 hpi (10^2.010±0.189^ TCID_50_/mL), and the virus titre increased during the following hours and peaked at 48 hpi (10^6.196±0.229^ TCID_50_/mL, Figure 1). The results demonstrate that SIV (A/Swine/Shandong/3/2005) retains the ability to infect and replicate in primary cultured porcine alveolar macrophages.

### Kinetics of mRNA expression of receptors, signaling proteins and effector molecules

TLR-3 and TLR-7 mRNA expression increased after the cells were exposed to virus.

**Figure 2:**
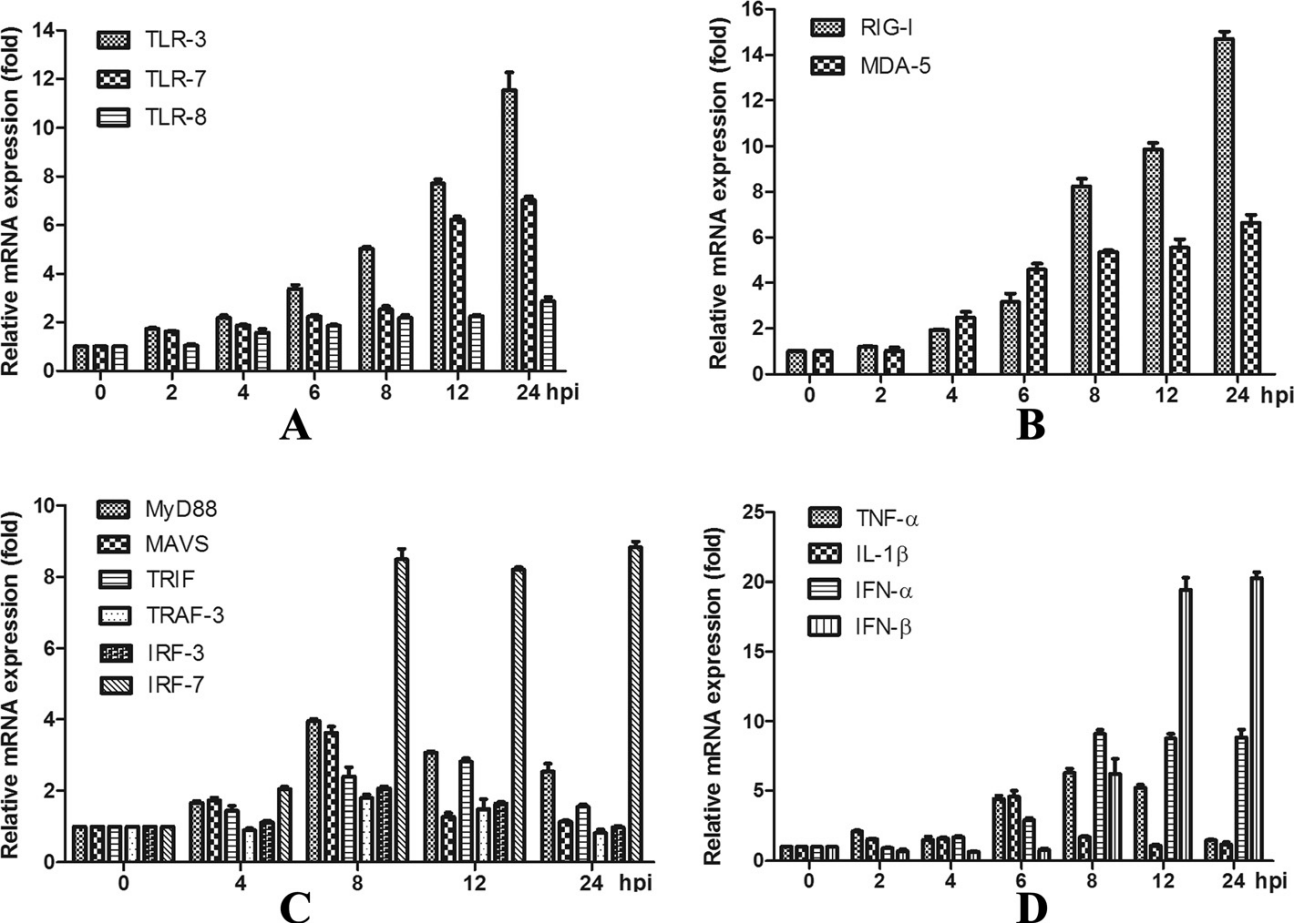
Kinetics of mRNA expression of receptors (TLR-3, TLR-7, TLR-8, RIG- I and MDA5) **(** **A** **,** **B** **)**, signaling proteins (MyD88, MAVS, TRIF, TRAF-3, IRF-3, and IRF-7) **(** **C** **)** and effector molecules (TNF-α, IL-1β, IFN-α and IFN-β) **(** **D** **)** after virus infection. PAMs were infected with SIV (A/Swine/Shandong/3/2005) at a multiplicity of infection of 5. Mock-treated cells received virus-free culture medium. Macrophages were harvested for RNA isolation at 0, 2, 4, 6, 8, 12, and 24 hpi. Data are presented as means ± SEM (n = 3). Results are representative of 3 independent experiments.

Greater than 6-fold changes were observed at 12 hpi and 24 hpi (P < 0.05) after H3N2 infection compared to the control group. There was mild TLR-8 mRNA expression after H3N2 challenge. Two fold increases were observed at 8, 12 and 24 hpi (P < 0.05) (Figure 2A).

H3N2 infection significantly increased mRNA expression of RIG-I after 4 hpi (P *<* 0.05). More than 6-fold increases were present at 8, 12, and 24 hpi (8.240 ± 0.34, 9.86 ± 0.28 and 14.7 ± 0.33, respectively) (P < 0.05). MDA5 mRNA expression also increased significantly after 4 hpi (P *<* 0.05). More than 4-fold increases were observed after 6 hpi (P < 0.05) (Figure 2B).

MyD88 mRNA expression peaked and a nearly 4-fold increase was present at 8 hpi (3.96 ± 0.055) (P < 0.05). MAVS mRNA expression decreased at 2 hpi (0.59 ± 0.03), increased during the following hours and peaked at 8 hpi (3.62 ± 0.18). TRIF mRNA expression increased after SIV challenge and reached its highest level at 12 hpi (2.81 ± 0.09). H3N2 challenge decreased TRAF-3 mRNA expression during the first few hours after which expression increased. Significant decreases were present at 2 hpi (0.69 ± 0.13) (P < 0.05) and a significant increase was present at 8 hpi (1.79 ± 0.1) (P < 0.05). A significant change (P *<* 0.05) in IRF-3 mRNA expression was present at 8 hpi compared to controls. However, only a 2.1-fold increase was detected. IRF-7 mRNA expression increased significantly from 2 hpi (1.98 ± 0.1) (P < 0.05) and more than 8-fold increases were observed after 8 hpi (P < 0.05) (Figure 2C).

H3N2 infection increased the expression of TNF-α mRNA in PAMs. More than 4-fold increases were present at 6, 8 and 12 hpi (4.4 ± 0.25, 6.29 ± 0.29 and 5.21 ± 0.21, respectively) (P < 0.05). IL-1β mRNA expression was mild and a 4.6 fold increase was observed at 6 hpi. IFN-α mRNA expression increased from 4 hpi (P *<* 0.05) compared to controls and peaked at 8 hpi (9.1 ± 0.29) (P < 0.05). Significant increases (P < 0.05) of IFN-β mRNA expression were observed at 8, 12 and 24 hpi (6.22 ± 1.09, 19.44 ± 0.84 and 20.27 ± 0.41, respectively) (Figure 2D).

### Expression of RIG-I, TLR-3, TLR-7, MAVS and MyD88 in PAMs

**Figure 3:**
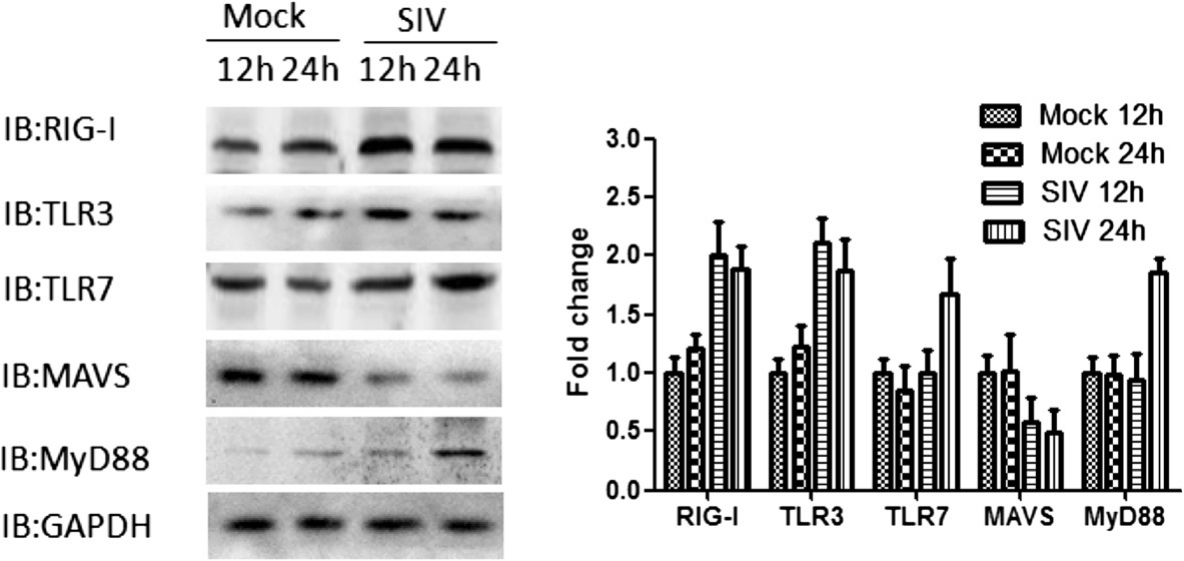
Changes in protein expression of RIG- I, TLR-3, TLR-7, MAVS, MyD88 after virus infection. PAMs were infected with SIV (A/Swine/Shandong/3/2005) at a multiplicity of infection of 5. Mock-treated cells received virus-free medium. Cells were harvested at 12 and 24 hpi. Data are presented as means ± SEM (n = 3). Results are representative of 3 independent experiments.

The expression of RIG-I and TLR-3 proteins increased after SIV infection. A 2 fold increase was observed at 12 hpi and more than a 1.5-fold increase at 24 hpi compared to mock infected controls for these two receptors. Significant changes for TLR-7 and MyD88 protein expression were detected at 24 hpi. Compared to mock infected controls, the expression of MAVS protein levels significantly decreased after SIV challenge (Figure 3).

### Changes of TNF-α, IL-1β, IFN-α and IFN-β levels

**Figure 4:**
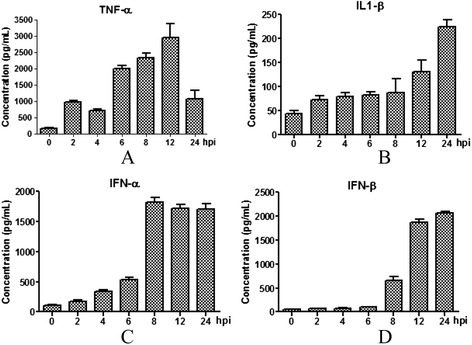
Concentration kinetics of TNF-α **(** **A** **)**, IL-1β **(** **B** **)**, IFN-α **(** **C** **)** and IFN-β **(** **D** **)** after virus infection. PAMs were infected with SIV (A/Swine/Shandong/3/2005) at a multiplicity of infection of 5. Mock-treated cells received virus-free medium. Culture supernatants were harvested at 0, 2, 4, 6, 8, 12, and 24 hpi. Data are presented as means ± SEM (n = 3). Results are representative of 3 independent experiments.

TNF-α concentration peaked at 12 hpi (2937.41 ± 435.85 pg/mL) after H3N2 challenge (P < 0.05), approximately 20-fold higher than at 0 h (147.39 ± 29.27 pg/mL). IL-1β level peaked at 24 hpi (224.77 ± 14.20 pg/mL) (P < 0.05). IFN-α increased significantly after H3N2 infection and more than 16-fold increases were present at 8, 12 and 24 hpi (1819.10 ± 76.23 pg/mL, 1716.70 ± 61.42 pg/mL and 1712.94 ± 79.42 pg/mL, respectively) (P < 0.05). Significant differences in IFN-β levels were observed at 8, 12 and 24 hpi (653.46 ± 71.54 pg/mL, 1868.10 ± 60.04 pg/mL and 2054.90 ± 42.42 pg/mL, respectively) compared to the 0 h control (Figure 4).

## Discussion

Macrophages reside beneath the respiratory epithelium and play a critical role in the initiation of immune responses against pathogens, including influenza viruses. During influenza viral replication in bronchial epithelial cells, macrophages are one of the earliest targets infected [17]. In the current study, PAMs were obtained from porcine lungs and cultured *in vitro*. The results showed that H3N2 SIV could infect and replicate in PAMs and that virus titers reached 10^6.196±0.229^ TCID_50_/mL at 48 hpi.

Viral infection results in the activation of multiple signaling pathways. Among them, TLRs and RLRs mediated signaling pathways play key roles in response to RNA virus infection [9-11]. Herein, we quantified the changes in these related receptors, signaling proteins and effector molecules post H3N2 virus infection of PAMs that express both TLRs and RLRs.

TLRs are transmembrane signaling proteins present in most cell types that recognize various proteins, carbohydrates, lipids and nucleic acids of invading microorganisms [10]. To date, 13 members of the TLR family have been identified [10,22]. The genome of H3N2 SIV, a ssRNA virus, is rich in uridine or uridine/guanosine that is mainly detected by TLR-7 and/or TLR-8 [23]. In the current study, the levels of TLR-7 mRNA expression increased after the cells were exposed to virus. Greater than 6-fold changes were observed at 12 and 24 hpi. There was mild TLR-8 mRNA expression after H3N2 SIV challenge; only a 2 fold maximal increase was observed. A previous study documented that, though the functions of TLR-7 and TLR-8 are closely related, their responsiveness to ssRNA may not be equal among animal species [24,25]. Both TLR-7 and TLR-8 can sense ssRNA in human systems whereas only TLR-7 functions in murine systems [23,26].

TLR-7 significantly increased at 24 hpi compared to mock infected controls. These findings are consistent with a previous study with a different H3N2 influenza A virus where TLR-7 activation enhanced the NOX2 oxidase-dependent oxidative burst in macrophages, which may underpin the acute lung injury in influenza A virus infection [27]. TLR-3 may play a central role in host responses to viral infections. TLR-3 mainly detects dsRNA (also ssRNA, such as in West Nile Virus). Since dsRNA can be produced during replication of influenza viruses, we presume that TLR-3 has an important role in H3N2 infection. In the current study, mRNA and protein expression of TLR-3 increased at 12 and 24 hpi.

It is well known that following receptor/ligand interaction, both TLR-7 and TLR-8 can recruit the universal TLR adapter protein MyD88, which functions downstream of all TLRs except TLR-3 [22,28]. Subsequently, the PRR-triggered signal passes in the cytoplasm through a MyD88-dependent pathway eventually resulting in the activation of NF-κB. The activated NF-κB diffuses into the nucleus and causes a strong transcription response subsequently inducing the secretion of inflammatory cytokines [10,29]. Our results show that MyD88 proinflammatory cytokines (TNF-α and IL-1β) and transcriptional and translational products, increase after infection. A 3-fold increase was observed in MyD88 mRNA expression and more than a 4-fold increase was induced in TNF-α and IL-1β. Western blotting analysis showed that significant changes in MyD88 protein expressions were detected at 24 hpi. The concentrations of TNF-α and IL-1β peaked at 12 and 24 hpi. These data suggest that, in swine macrophages, protein expression in the TLR-7 and/or TLR-8-MyD88 axes are altered as a result of H3N2 influenza virus infection and these axes may be responsible for the anti-virus response. TLR-3 induces inflammatory cytokines through a TRIF-dependent pathway. In the present study, TRIF mRNA increased after influenza virus challenge. Thus, TLRs (TLR-3, TLR-7/8) -induced inflammatory cytokines may be important in SIV infection. The central roles of the corresponding signaling pathways in host antivirus activation have been established for other influenza virus subtypes [30].

The signaling pathways mediated by RLRs may be critical in host cells detecting and responding to virus infection. RIG-I was discovered in 1997 (GenBank: AF038963) and later described in pigs as a helicase induced by infection with porcine reproductive and respiratory syndrome virus [31]. It has been established that RIG-I can be activated by diverse viruses including influenza, hepatitis C and others. Through an E3-ligase tripartite motif-containing 25 (TRIM25)-dependent pathway, ubiquitination of RIG-I occurs. This promotes its interaction with MAVS (MAVS is a pivotal cellular antiviral protein whose expression directly determines antiviral ability of the host cell) that recruits RIG-I and MDA5 to the mitochondrial outer membrane as part of a macromolecular signaling complex. This triggers the activation of IRF-3/7 and NF-κB transcription factors that in turn induce robust type I interferons and proinflammatory cytokines [32,33]. The importance of RIG-I has been demonstrated in experimental models of infection with respiratory viruses in RIG-I-deficient cells [34]. In the experiments described herein, H3N2 SIV infection significantly elevated mRNA and protein expression of RIG-I. Although MDA5 and RIG-I may share similar signaling pathways and structural homologies, the two helicases may discriminate among different ligands that trigger innate immune responses to RNA viruses [35,36]. We detected MDA5 mRNA expression after H3N2 SIV challenge. Similar changes with RIG-I expression were observed in MDA5 mRNA expression post-infection. These data prove that H3N2 SIV infection can regulate the synthesis of RIG-I and MDA5 which sequentially activates the signaling pathways mediated by them in porcine macrophages. This conclusion is reinforced by the detection of downstream adaptor proteins and effector molecules. The expression of MAVS, IRF-3/7, and type I interferons (IFN-α and IFN-β) are all altered post-infection. In this study, compared to mock, MAVS protein levels significantly decreased after SIV challenge. The result is consistent with a previous study demonstrating that RLR activation triggers MAVS ubiquitination on lysine 7 and 10 by the E3 ubiquitin ligase TRIM25 and marks it for proteasomal degradation concomitant with downstream signaling. While, inhibition of MAVS degradation with a proteasome inhibitor hampers IRF-3 activation [37].

The rapid production of type I interferons is a central and essential step in the induction of interferon-stimulated genes (ISGs) whose products direct antiviral and immunomodulatory actions that can limit infection [38]. In addition to RIG-I/MDA5-MAVS-IRFs signaling pathways, TLRs-MyD88/TRAF3-IRFs are believed to be critical for the production of type I interferons. Häcker et. al. using myeloid cells from TRAF3- and TRAF6-deficient mice, discovered that TRAF3 binds MyD88 and IRAK1, thereby activating IRF-7 and inducing type I IFN [39]. In the current study, H3N2 SIV challenge decreased TRAF-3 mRNA expression during the first few hpi after which expression increased. Significant decreases were present at 2 hpi; a significant increase was observed at 8 hpi. IFN-α mRNA and protein levels increased after SIV infection and significant differences in IFN-β mRNA and protein expression was observed at 8, 12 and 24 hpi. This indicates that TRAF3 is critical for the induction of type I IFN in the swine macrophage response to H3N2 SIV challenge. In research of innate signaling pathways, it is important to use specific inhibitors or siRNA to establish the dominant signaling mechanisms that promote anti-viral and proinflammatory cytokine production. We made numerous attempts to introduce siRNA oligos (such as siTRAF3, siIRF3 or siIRF7) or the non-degradable super-repressor form of IκBα (which is a known potent inhibitor of NF-κB) into primary cultured porcine alveolar macrophages. However, the primary cells were much more difficult to transfect than more commonly used HEK293 or A549 cell lines. Because of the lower efficiencies of transfection, there were no significant results. Additionally, in the current study, we focused on TRAF3, IRF-3 and IRF-7 mediated signaling pathways that induce cytokine expression. Cytokine production through the TRAF6, NF-κB pathways are important in host antivirus activity [40,41]. The efficiency of transfection and the role of TRAF6, NF-κB in host antivirus activity need to be further studied.

## Conclusion

The results of this study showed that the H3N2 influenza strain infection significantly influenced the expression of the receptors, adapter proteins and downstream effector molecules of RLRs and TLRs signaling pathways. RLRs and TLRs mediated innate immunity signaling pathways are important in the ability of PAMs to respond to H3N2 SIV infection. This study provides a better understanding of innate immunity signaling pathways in PAM activity against H3N2 SIV infection.
